# Highly Efficient White Organic Light-Emitting Diodes with Ultrathin Emissive Layers and a Spacer-Free Structure

**DOI:** 10.1038/srep25821

**Published:** 2016-05-12

**Authors:** Shengfan Wu, Sihua Li, Qi Sun, Chenchao Huang, Man-Keung Fung

**Affiliations:** 1 Institute of Functional Nano & Soft Materials (FUNSOM), Jiangsu Key Laboratory for Carbon-Based Functional Materials & Devices, Soochow University, 199 Ren’ai Road, Suzhou, 215123, Jiangsu, PR China

## Abstract

Ultrathin emissive layers (UEMLs) of phosphorescent materials with a layer thickness of less than 0.3 nm were introduced for high-efficiency organic light-emitting diodes (OLEDs). All the UEMLs for white OLEDs can be prepared without the use of interlayers or spacers. Compared with devices fabricated with interlayers inserted in-between the UEMLs, our spacer-free structure not only significantly improves device efficiency, but also simplifies the fabrication process, thus it has a great potential in lowering the cost of OLED panels. In addition, its spacer-free structure decreases the number of interfaces which often introduce unnecessary energy barriers in these devices. In the present work, UEMLs of red, green and blue-emitting phosphorescent materials and yellow and blue phosphorescent emitters are utilized for the demonstration of spacer-free white OLEDs. Upon optimization of the device structure, we demonstrated spacer-free and simple-structured white-emitting OLEDs with a good device performance. The current and power efficiencies of our white-emitting devices are as high as 56.0 cd/A and 55.5 lm/W, respectively. These efficiencies are the highest ever reported for OLEDs fabricated with the UEML approach.

Since C.W Tang demonstrated the first organic light-emitting diodes (OLEDs) based on a double-layer structure of organic materials in 1987 1, flat-panel displays and lighting applications based on OLED technology have grown dramatically because of their attractive features such as simple fabrication process, ultra-thin structure, light-weight and flexibility^2^,^3^,^4^,^5^,^6^,^7^,^8^,^9^,^10^,^11^,^12^. It was also found that phosphorescent emissive materials can harvest both singlet and triplet excitons and therefore an internal quantum efficiency of 100% can be obtained in OLEDs^13^,^14^,^15^,^16^,^17^,^18^. In particular, white-emitting OLEDs are known to be an ideal light source without “blue” hazard, which not only function as a backlight for OLED display and an area light source for decorative and general lightings, but can also be applied as lighting in galleries, hospitals and museums because OLED has no ultraviolet emission.

Currently, one of the major challenges for OLED commercialization is its cost, in which organic materials constitute approximately 20% of the total cost in a panel^19^. One way to reduce the manufacturing cost is to simplify the fabrication process. It is common that in order to achieve a desirable OLED performance, a host-dopant system is adopted. It is therefore crucial to select a host whose energy level aligns well with the dopants resulting in an efficient energy transfer. However, in reality there are few host materials which can match red and blue emitters at the same time. As a consequence, the device structure of OLED is rather complicated, typically consisting of 2 to 3 host-dopant systems. This not only leads to an increase in material cost and device processing time, but also makes it difficult to control dopant concentration accurately. As such, a number of studies have been reported with simplified device structures. Wang *et al.*^20^. fabricated high-efficiency and good color-stability white OLEDs by using a single host. Sun *et al.*^21^ reduced the number of organic layers by removing the interlayer between the fluorescent and phosphorescent materials in hybrid white OLEDs.

Recently, OLEDs with dopant-free and ultrathin emissive layers (UEMLs) have aroused much attention^22^,^23^,^24^,^25^,^26^,^27^,^28^,^29^,^30^,^31^,^32^. Chen *et al.*^23^ replaced conventional host-dopant systems with non-doped ultrathin bluish-green and red dyes to achieve high-efficiency white OLEDs. Tan *et al.*^24^. adopted the UEML approach to fabricate white OLEDs with an efficiency of 23.4 cd/A and 17.0 lm/W. Zhao *et al.*^25^. reported a dopant-free hybrid white OLEDs with ultrathin fluorescent blue and phosphorescent green and red emitters. The device has a current efficiency of 23.2 cd/A at a luminance of 1,000 cd/m^2^. The devices based on UEMLs have numerous merits compared with conventional host-guest systems. First, it is not necessary to consider energy level alignment between the hosts and dopants. Second, the dopant-free devices based on UEMLs should have a good color reproducibility. Third, the UEML approach may be able to save at least 70% of material cost, assuming 10 vol. % of expensive phosphorescent emitters doped in 10 nm thick hosts are replaced with UEMLs with a thickness of only 0.3 nm. In most of their work, interlayers or spacers were placed in between the UEMLs, which were used passively to tune the color balance and maximize the device efficiency. Nevertheless, the interlayers would introduce new interfaces which may cause a mismatch of energy level with neighboring UEMLs. The additional layers will also enhance the complexity of the devices.

In this paper, we first discuss high-efficiency red, green and blue-emitting OLEDs based on phosphorescent UEMLs, the efficiencies of which are comparable or even better than those fabricated using conventional host-guest systems. Simple and high-efficiency white OLEDs were also fabricated using phosphorescent UEMLs consisting of red, green and blue emitters as well as orange and blue emitting materials, without any interlayer. The color balance was actively tuned by the thickness of the UEMLs in a much simpler and more controllable way. Therefore, UEMLs with spacer-free structures have a great potential in achieving power-efficient white OLEDs.

## Results and Discussion

**Figure 1:**
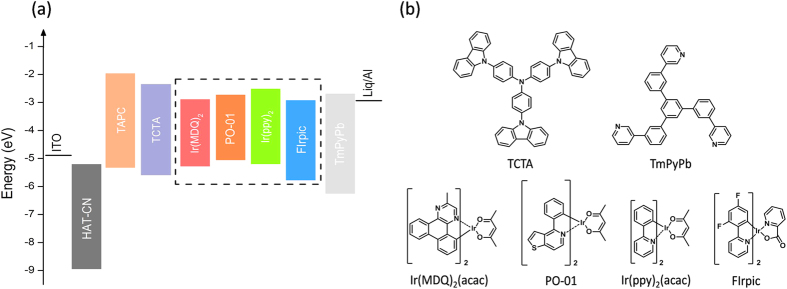
(**a**) Energy band diagram and device structures with different UEMLs, and (**b**) Chemical structures of different transport and emissive materials.

**Figure 2:**
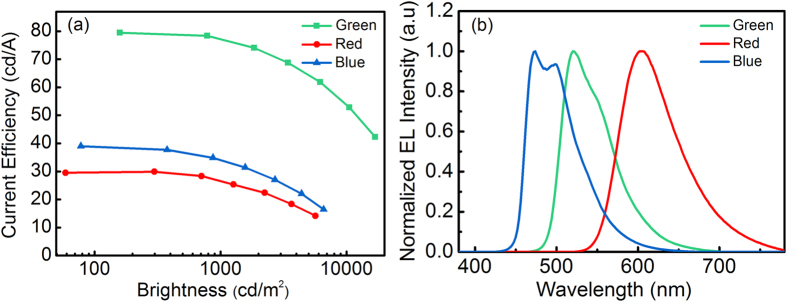
(**a**) Current efficiency–brightness characteristics, and (**b**) Normalized EL spectra of the red, green and blue-emitting devices based on ultrathin Ir(MDQ)_2_(acac), Ir(ppy)_2_(acac), and FIrpic. Each UEML is 0.3 nm thick.

The device structures of monochrome OLEDs were: ITO/HAT-CN (10 nm)/TAPC (45 nm)/TCTA (10 nm)/UEML (0.3 nm)/TmPyPb (40 nm)/Liq (2 nm)/Al (120 nm), where the UEML was Ir(MDQ)_2_(acac), Ir(ppy)_2_(acac) or FIrpic. Figure 1a depicts the schematic energy band diagram of OLED structures with different phosphorescent UMELs, namely Ir(MDQ)_2_(acac), PO-01, Ir(ppy)_2_(acac) and FIrpic, with red, orange, green and blue emission, respectively. Their chemical structures are shown in Fig. 1b. It can be deduced from their energy levels and carrier-transport properties of each layer that the exciton recombination zone of the device is located at the interface between TCTA and TmPyPb because most of the holes will pile in the vicinity of the TCTA/TmPyPb interface owing to the high hole-injection barrier between the highest occupied molecular orbitals (HOMO) of TCTA (HOMO = 5.80 eV) and TmPyPb (HOMO = 6.75 eV). Since TCTA has a relatively poor electron transport property, which merely has an electron-mobility of 10^−8^  cm^2^/Vs^33^, it is difficult for electrons to be injected from TmPyPb into TCTA although the electron-injection barrier between the lowest unoccupied molecular orbitals (LUMO) of TmPyPb (LUMO = 2.75 eV) and TCTA (LUMO = 2.40 eV) is merely 0.35 eV. Therefore, TCTA and TmPyPb should be a good confinement zone for excitons. Fig. 2 depicts the device performance and electroluminescent spectra of red, green and blue PHOLEDs with the UEML deposited in between the TCTA and TmPyPb. It can be seen from Fig. 2a that the red, green and blue devices exhibit a maximum current efficiency of 29.9 cd/A, 79.5 cd/A and 39.0 cd/A, respectively, while the external quantum efficiencies (EQE) of the three devices are 16.2%, 21.1% and 17.6% accordingly. All the device performance as shown above indicates that the exciton energies were absorbed by the UMELs effectively^29^.

In order to express the promising potential of UEMLs, several green-emitting phosphorescent OLEDs (PHOLEDs) with different conventional host-guest systems, namely CBP:Ir(ppy)_2_(acac), TmPyPb:Ir(ppy)_2_(acac) or TCTA:Ir(ppy)_2_(acac), were fabricated for comparison. The doping concentration was fixed at 10%, and the device structures are as follows:

Device G1: ITO/HAT-CN (10 nm)/TAPC (45 nm)/TCTA (10 nm)/CBP:10% Ir(ppy)_2_(acac) (20 nm)/TmPyPb (40 nm)/Liq (2 nm)/Al (120 nm).

Device G2: ITO/HAT-CN (10 nm)/TAPC (45 nm)/TCTA (2 nm)/TCTA:10% Ir(ppy)_2_(acac) (8 nm)/TmPyPb (40 nm)/Liq (2 nm)/Al (120 nm).

Device G3: ITO/HAT-CN (10 nm)/TAPC (45 nm)/TCTA (10 nm)/TmPyPb:10% Ir(ppy)_2_(acac) (8 nm)/TmPyPb (32 nm)/Liq (2 nm)/Al (120 nm).

**Figure 3:**
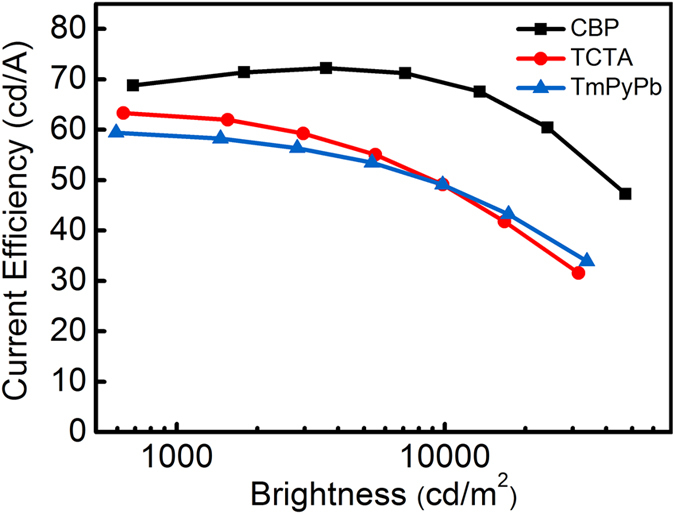
Current efficiency–brightness characteristics of different doped green-emitting OLEDs based on Ir(ppy)_2_(acac) as a dopant (a doping ratio of 1:10), and either CBP, TCTA or TmPyPb as a host.

It can be seen from the current efficiency – brightness characteristics in Fig. 3 that Device G1 using CBP as a host has a highest maximum current efficiency of 72.2 cd/A, while Devices G2 and G3 have relatively lower efficiencies of 63.3 cd/A and 59.4 cd/A, respectively. CBP is a better host because of its high triplet energy resulting from the small singlet-triplet splitting^34^,^35^,^36^. The above results indicate that the host materials have a significant influence on device performance, and the choice of host material is of great importance. Table 1 summarizes the device performance of different green-emitting PHOLEDs. It is remarkable that the performance of green OLED prepared by the UEML approach surpasses those fabricated by the conventional host-guest systems. In particular, in comparison with the prototypical green PHOLED comprising CBP:Ir(ppy)_2_(acac), the current efficiency, power efficiency and EQE of the UEML-based device can be increased by 10%, 40% and 17%, respectively. Another merit of using UEML is that it is not necessary to select host materials for a specific dopant. After all, it is difficult to find a suitable blue host material with a sufficiently high triplet energy and high thermal stability for blue PHOLED.

Compared with the UEML-based OLED, PHOLED has relatively reduced efficiency roll-off. It is because the excitons formed in the ultrathin layer of phosphorescent emitter (less than 0.3 nm thick) may have a higher triplet density than those formed in the conventional guest-host PHOLED where the excitons are able to disperse within the host materials. Thus, the UEML-based device may have higher probability of triplet–triplet annihilation (TTA) and hence faster efficiency roll-off than conventional OLEDs. However, in our case the efficiency roll-off is not too severe. The current efficiency drops about 10% from its maximum value for a luminance at 3,000 cd/m^2^ which is the brightness generally required for commercial applications. To further improve the efficiency roll-off problem, we can consider to insert a layer of host molecule such as mCP, CBP, etc. or bipolar host material, in between the UMEL so as to reduce the exciton density on the emitters, thus alleviating the TTA and reducing the efficiency roll-off.

In view of the above remarkable results, two-color PHOLEDs based on green and blue UEMLs were fabricated. For those multi-color OLEDs currently prepared with the UEML approach, an interlayer is usually inserted in between two emissive layers. However, ultrathin sheets of dye molecules with a thickness of less than 0.5 nm do not form a neat layer^32^. Instead, the molecules may just scatter on the surface of the underlying organic films, and there are probably many unoccupied sites on the surface. When the second ultrathin layer of dyes is deposited on the surface of the first one without any interlayer, it can be speculated that the second UEML may fill the unoccupied sites randomly as an interstitial doping. Therefore, interlayer or spacer may not be necessary in multi-color UEML based devices.

**Figure 4:**
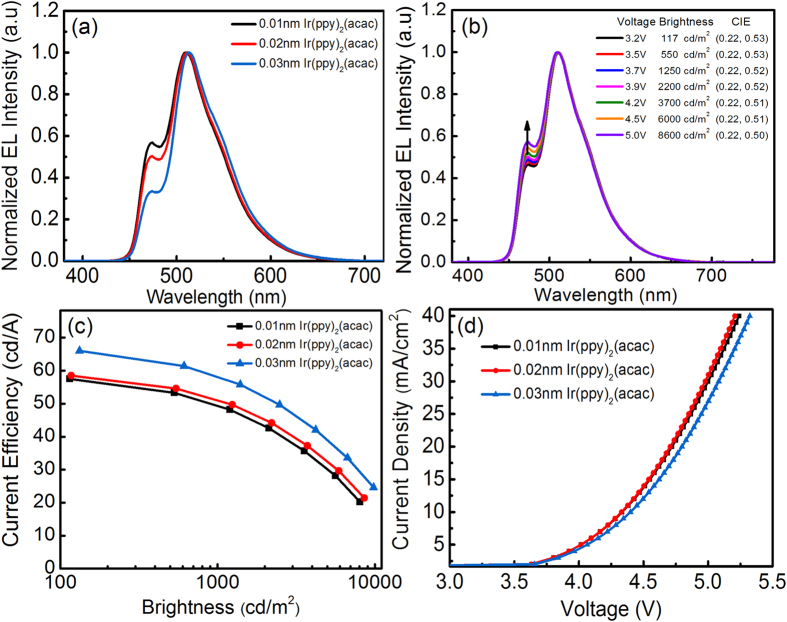
(**a**) EL spectra of the devices based on three different thickness of Ir(ppy)_2_(acac), (**b**) Evolution of EL spectra with increasing driving voltage, (**c**) Current efficiency–brightness characteristics, and (**d**) J-V curves of the devices.

To demonstrate simple and spacer-free UEML-based OLEDs, devices with the following structures were fabricated: ITO/HAT-CN (10 nm)/TAPC (45 nm)/TCTA (10 nm)/ Ir(ppy)_2_(acac) (X nm)/FIrpic (0.3 nm)/TmPyPb (40 nm)/Liq (2 nm)/Al (120 nm), where X was 0.01 nm, 0.02 nm and 0.03 nm, respectively. Figure 4a shows the EL spectra of the bluish-green devices as a function of Ir(ppy)_2_(acac) thickness. With increasing Ir(ppy)_2_(acac) thickness, the blue emission gradually decreased. Therefore, the key step to optimize the color balance of multi-color UEML-based devices with an interlayer free structure is to simply modulate the thickness of the UEMLs which have a lower T_1_ or S_1_. There are two scenarios to explain the emission mechanisms. First, the bluish-green emission is due to exciton diffusion. The triplet energy levels (T_1_) of FIrpic and Ir(ppy)_2_(acac) are 2.65 eV and 2.47 eV, respectively^37^,^38^. Therefore the excitons generated between TCTA and TmPyPb immediately migrate and transfer the energy to Ir(ppy)_2_(acac) prior to being transferred to FIrpic. When the thickness of Ir(ppy)_2_(acac) was only 0.01 nm, the green emission was saturated with triplet excitons easily and hence more excitons migrated to FIrpic for blue emission. If the thickness of the green dyes increased, more excitons will be consumed by the Ir(ppy)_2_(acac) rather than the FIrpic, resulting in a decrease of blue emission. Second, the bluish-green emission may be due to Forster transfer since their distances were spatially located within the Forster radii^24^. Figure 4b shows the variation of EL spectra, brightness and CIE with increasing driving voltage for the device consisting of 0.02 nm thick of Ir(ppy)_2_(acac). It is observed that the blue emission increased with driving voltage, indicating that the emission mechanism as discussed above is due to exciton diffusion. In such a case, increasing voltage will facilitate exciton migration from the green to blue emitters. It should also be noted in Fig. 4b that the color was quite stable for brightness increasing to over 8,000 cd/m^2^.

In addition, it can be seen in Fig. 4c that the current efficiency increased from 51 cd/A to 58 cd/A at a brightness of 1,000 cd/m^2^ when the thickness of Ir(ppy)_2_(acac) increased from 0.02 nm to 0.03 nm. The increase in efficiency is simply attributed to a shift to a more greenish emissive color. Figure 4d shows the J-V characteristics of the devices. The J-V curves are almost identical for devices with a thickness of Ir(ppy)_2_(acac) altering from 0.01 nm to 0.02 nm. However, when the thickness of Ir(ppy)_2_(acac) increased to 0.03 nm, the current density decreased for a driving voltage higher than 4 V. This suggests that there were two regimes. When the thickness of the green dyes was less than 0.02 nm, the ultrathin green-emitting layer had negligible effect on the transport properties of the devices. It is a straightforward exciton diffusion process from Ir(ppy)_2_(acac) to FIrpic within the narrow exciton recombination zone. If the thickness of the green dyes was increased, the excessive luminescent sites would become trap states for excitons and therefore carrier trapping also plays a dominant role in the emission mechanism.

**Figure 5:**
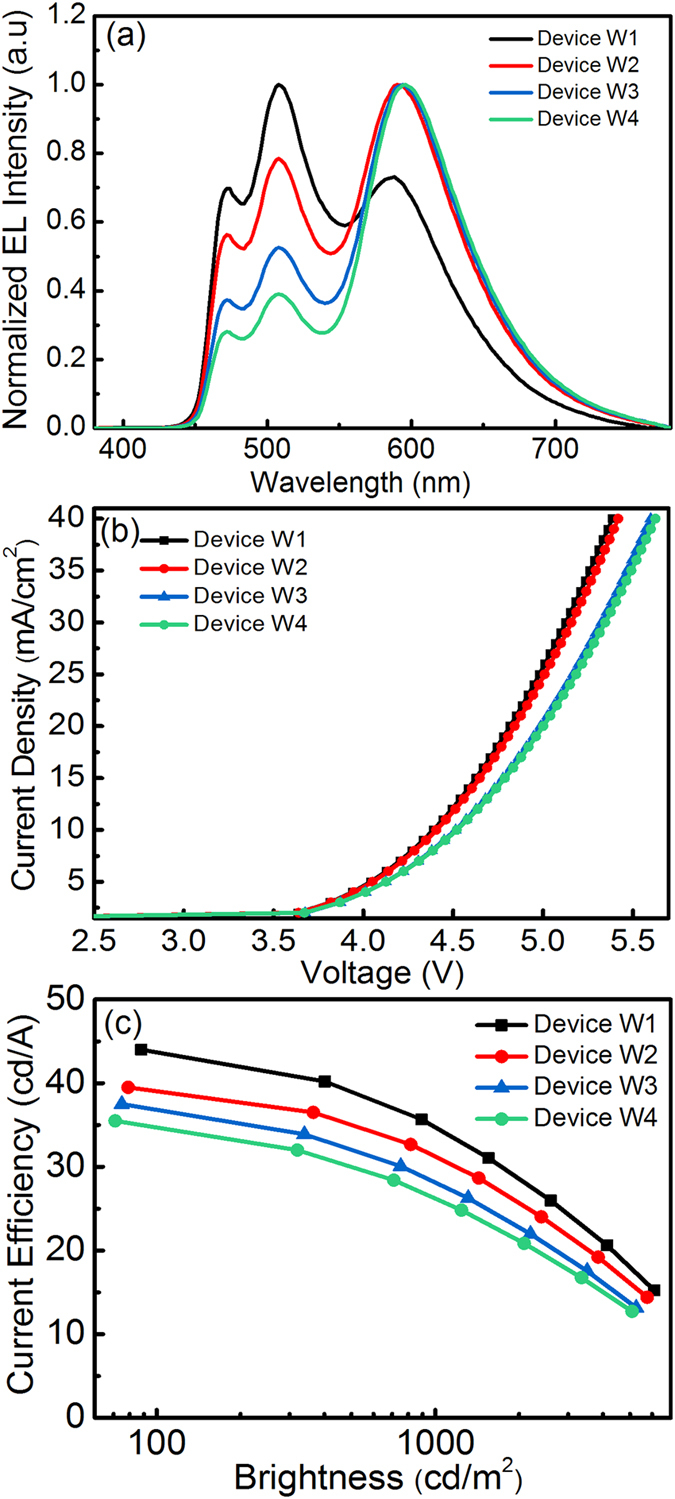
(**a**) EL spectra of the devices based on different thicknesses of Ir(MDQ)_2_(acac) varying from 0.02 nm (W1), 0.04 nm (W2), 0.06 nm (W3) and 0.08 nm (W4), (**b**) J-V curves, and (**c**) Current efficiency–brightness characteristics of the devices.

Once the thickness of Ir(ppy)_2_(acac) has been optimized, three-color white OLEDs based on red, green and blue UEMLs were explored. The device structure is: ITO/HAT-CN (10 nm)/TAPC (45 nm)/TCTA (7 nm)/ Ir(ppy)_2_(acac) (0.02 nm)/FIrpic (0.3 nm)/TCTA (3 nm)/ Ir(MDQ)_2_(acac) (Y nm)/TmPyPb (3 nm)/Ir(ppy)_2_(acac) (0.02 nm)/FIrpic (0.3 nm)/TmPyPb (37 nm)/Liq (2 nm)/Al (120 nm), where Y = 0.02 nm (device W1), 0.04 nm (device W2), 0.06 nm (device W3), and 0.08 nm (device W4). It is obvious that the exciton generation zone is located at the interface between TCTA and TmPyPb. The triplet energy level of Ir(MDQ)_2_(acac) is much lower than the counterpart of FIrpic (T_1 _= 2.65 eV) and Ir(ppy)_2_(acac) (T_1_ = 2.47 eV). Ir(MDQ)_2_(acac) has a narrow band gap, i.e., deep LUMO energy level and shallow HOMO energy level, and thus charge trapping would happen easily. It was placed at the interface of TCTA/TmPyPb, which acts as a deep trap for triplet exciton. The EL spectra in Fig. 5a clearly shows three emissions with peaks at 470 nm, 510 nm and 610 nm, which originated from FIrpic, Ir(ppy)_2_(acac) and Ir(MDQ)_2_(acac), respectively. When the thickness of Ir(MDQ)_2_(acac) was 0.02 nm, the red dyes were saturated with triplet excitons quickly and the rest of excitons migrated to both the TCTA/TCTA and TmPyPb/TmPyPb interfaces for green and then blue emissions. This is the reason why green and blue emissions are stronger than red. It is interesting to note that blue and green emission intensity was dramatically reduced when the thickness of Ir(MDQ)_2_(acac) was increased from 0.02 nm to 0.08 nm. In such a case, some excitons from the red dyes could be trapped, which reduced the number of triplet excitons being transferred to the green and blue dyes. Our hypothesis is confirmed by the J-V characteristics as shown in Fig. 5b. It is observed that the current density obviously decreased as expected for devices W3 and W4, implying the charge-trapping effect.

Figure 5c shows the device performance of W1 to W4, and it can be seen that the maximum current efficiency of Devices W1, W2, W3 and W4 are 44 cd/A, 39.5 cd/A, 37.5 cd/A and 35.5 cd/A, respectively. Table 2 summarizes the device performance of different white OLEDs. All the devices have a very low turn-on voltage of 3.2 V (defined at a current density of 0.2 mA/cm^2^). It is remarkable that the white OLED (Device W2) which exhibits an CIE (x, y) of (0.43, 0.44) has current and power efficiencies of 39.5 cd/A and 38.9 lm/W, respectively. If the white OLED had a warmer color (Device W1), we could achieve current and power efficiencies of 44.0 cd/A and 43.1 lm/W, respectively. Note that all the devices have no light out-coupling films.

**Figure 6:**
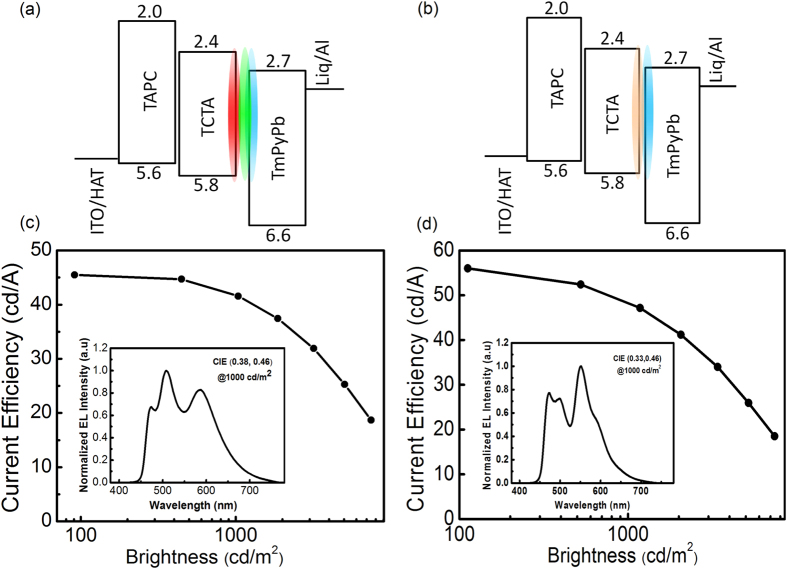
(**a**) Device structure of the three-color white OLED based on ultrathin red, green and blue emitters, which are 0.02 nm, 0.02 nm and 0.3 nm thick, respectively, (**b**) Device structure of the two-color white OLED based on ultrathin orange and blue emitters, which are 0.02 nm and 0.3 nm, respectively, (**c**) and (**d**) are the current efficiency–brightness characteristics and EL spectra (inset) of W5 and W6, respectively.

To further simplify the device structure and take full advantage of UEMLs, three-color (Device W5) and two-color (Device W6) white OLEDs without interlayers were demonstrated. The device structure of Device W5 and Device W6 are shown in Fig. 6a,b, which were ITO/HAT-CN (10 nm)/TAPC (45 nm)/TCTA (10 nm)/Ir(MDQ)_2_(acac) (0.02 nm)/Ir(ppy)_2_(acac) (0.02 nm)/FIrpic (0.3 nm)/TmPyPb (40 nm)/ Liq (2 nm)/Al (120 nm) (Device W5) and ITO/HAT-CN (10 nm)/TAPC (45 nm)/TCTA (10 nm)/PO-01 (0.02 nm)/FIrpic (0.3 nm)/TmPyPb (40 nm)/Liq (2 nm)/Al (120 nm) (Device W6), respectively. Fig. 6c,d show their device performance and EL spectra at a brightness of 1,000 cd/m^2^. The maximum current efficiency of device W5 is as high as 45.5 cd/A. It can also be seen in Table 2 that W5 has a turn-on voltage, power efficiency and EQE of 3.2 V, 46.1 lm/W and 17.6%, respectively. The two-color white OLED (Device W6) has an even better device performance. The ultrathin dyes are PO-01 and FIrpic, with respective thicknesses of 0.02 nm and 0.3 nm. It is observed from Fig. 6d that the current efficiency is nearly 50 cd/A at a brightness of 1,000 cd/m^2^. It can be seen in Table 2 that the two-color based device has a turn-on voltage, power efficiency and EQE of 3.1V, 55.5 lm/W and 19.3%, respectively. To our knowledge, these are the highest efficiencies reported so far for white OLEDs based on ultrathin emitting-layer.

## Conclusion

UEML based OLEDs show an excellent device performance. The red, green and blue OLEDs with UEML structures exhibit a current efficiency of 29.9 cd/A, 79.5 cd/A and 39.0 cd/A, respectively, which are comparable or even better than conventional OLEDs which use host-guest system. High-efficiency UEML based two-color and three-color white OLEDs have also been fabricated. It has been successfully demonstrated that interlayers or spacers are not needed, and the device structures of white OLEDs are significantly simplified. For three-color white OLEDs, current and power efficiencies of 39.5 cd/A and 38.9 lm/W could be obtained with CIE (x, y) of (0.43, 0.44). If the white OLED has a warmer emission (two-color white), the current and power efficiencies could be increased to 56.0 cd/A and 55.5 lm/W, respectively. The device efficiencies of our two-color and three-color white OLEDs demonstrated here are so far the highest ever reported for devices with ultrathin emitting-layer structures. Therefore, OLEDs fabricated with UEMLs are very cost-effective and promising.

## Methods

### Materials

Indium tin oxide (ITO) coated glass was used as a substrate in all the device studies. Dipyrazino [2,3-f:2′,3′-h] quinoxaline- 2,3,6,7,10,11-hexacarbonitrile (HAT-CN), 1,1-Bis-(4-methylphenyl)-aminophenyl)-cyclo-hexane (TAPC), N,N,N-tris(4-(9- carbazolyl)phenyl)amine (TCTA), 1,3,5-tri[(3-pyridyl)-phen-3-yl]benzene (TmPyPB), bis(2-methyldibenzo[f,h]-quinoxaline) (acetylacetonate) iridium(III) [Ir(MDQ)_2_(acac)], bis(4-phenylthieno[3, 2-c]pyridine) (acetylacetonate)iridium(III) (PO-01), bis(3,5-difluoro-2- (2-pyridyl)phenyl-(2-carboxypyridyl)iridium III (FIrpic), bis(2-phenylpyridine)(acetylacetonate)iridium(III) [Ir(ppy)_2_(acac)] and Lithium quinolate (Liq) were purchased from Xi’an Polymer Light Technology Corp. The purities of the materials are all over 99.9%.

### Device fabrication

Prior to device fabrication, the ITO substrate was ultrasonically cleaned sequentially with acetone, ethanol and deionized water, and dried in an oven at 120 °C for 5 h. It was then treated by ultraviolet ozone for 20 minutes before placing it in a vacuum chamber. HAT-CN was used as a hole-injection layer (HIL). TAPC and TCTA were applied as a hole-transport layer (HTL). TmPyPb and Liq were utilized as an electron-transport layer (ETL) and electron-injection layer (EIL), respectively. Ir(MDQ)_2_(acac), PO-01, Ir(ppy)_2_(acac) and FIrpic were phosphorescent red, orange, green and blue dopants respectively. All the materials were thermally evaporated through a shadow mask under a high vacuum of 2 × 10^−6^ Torr. The deposition rates of HAT-CN, TAPC, TCTA, TmPyPb, Liq and Al were 0.2, 2, 2, 2, 0.2, 7 Å/s, respectively. The deposition rate for UEMLs was controlled at 0.01–0.02 Å/s. The film thickness was accurately recorded by an Inficon thickness monitor with a rate resolution of 0.01 Å/s.

### Measurements

Electroluminescence (EL) spectra, current efficiency (CE), CIE coordinates and current density–voltage (J-V) characteristics were measured under a constant current source (Keithley 2400 s Source Meter) combined with a photometer (Photo Research PR 670 spectrophotometer). All the measurements were conducted in ambient air at room temperature, and the external quantum efficiency was calculated assuming Lambertian distribution of light emission.

## Additional Information

**How to cite this article**: Wu, S. *et al.* Highly Efficient White Organic Light-Emitting Diodes with Ultrathin Emissive Layers and a Spacer-Free Structure. *Sci. Rep.* **6**, 25821; doi: 10.1038/srep25821 (2016).

## Figures and Tables

**Table 1:** Summary of turn-on voltage, current efficiency, power efficiency and EQE of different green-emitting PHOLEDs.

Device	V_on_ (V)	Current efficiency (cd/A)_max_	Power efficiency (lm/W)_max_	External quantum efficiency (%)_max_
UEML	3.3	79.5	75.3	21.1
G1	3.8	72.2	54.1	18.1
G2	3.5	63.3	58.2	16.7
G3	3.5	59.4	53.5	15.5

**Table 2:** Summary of turn on voltage, CIE, current efficiency, power efficiency and EQE of the devices from W1 to W6.

Device	V_on_(V)	CIE^a^ (x, y)	Current efficiency (cd/A)		Power efficiency (lm/W)		External quantum efficiency (%)	
			CE^a^_max_	CE^b^	PE^a^_max_	PE^b^	EQE^a^_max_	EQE^b^
W1	3.2	(0.36, 0.45)	44.0	35.2	43.1	28.2	17.2	13.6
W2	3.2	(0.43, 0.44)	39.5	31.3	38.9	26.5	16.4	13.1
W3	3.2	(0.46, 0.43)	37.5	27.8	36.6	25.8	16.2	12.2
W4	3.2	(0.49, 0.43)	35.5	26.9	34.6	21.7	15.8	11.8
W5	3.1	(0.38, 0.46)	45.5	42.0	46.1	37.3	17.6	16.8
W6	3.2	(0.33, 0.46)	56.0	48.5	55.5	41.5	19.3	17.2

V_on_ is defined at a current density of 0.2 mA/cm^2^

CIE^a^ is the Commission Internationale de L’Eclairage (CIE) at a current density of 2.5 mA/cm^2^.

CE^a^_max,_ PE^a^_max_ and EQE^a^_max_ are the maximum current efficiency, maximum power efficiency and maximum external quantum efficiency.

CE^b^, PE^b^ and EQE^b^ are the current efficiency, power efficiency and external quantum efficiency at a brightness of 1,000 cd/m^2^.
